# Psychological features in lower risk melanoma: an analysis on 204 patients

**DOI:** 10.1186/1479-5876-12-S1-P4

**Published:** 2014-05-06

**Authors:** Ribero Simone, Bassino Stefania, Castelli Lorys, Grassi Marcella, Caliendo Virginia, Lauro Danilo, Balagna Elena Maira, Giaretti Marta, Leombruni Paolo, Torta Riccardo, Macripo Giuseppe

**Affiliations:** 1 University of Turin, Turin, Italy; 2 University of Turin, Neurosciences, Turin, Italy; 3 University of Turin, Psycology, Turin, Italy; 4 University of Turin, Medical Sciences, Turin, Italy

## Introduction & objectives

To describe psychological status in melanoma patients in a high risk distress moment: the follow up consultation. Secondly to know if there are any difference in distress and in other psychological variables between melanoma patients at stage Ib-II vs 0-Ia AJCC (American Joint Committed Cancer) and to correlate these with clinical variables.

## Material & methods

We recluted 204 continuous patients in 0-I-II melanoma stages during the follow-up visit from March to June 2011. They were submitted to a psychological interview and questionnaires (Distress Thermometer, Hospital Anxiety Depression Scale, Montgomery-Asberg Depression Rating Scale, SF-36, Brief cope). Patients were divided into two groups based on the stage of disease to achieve our second aim (0-Ia vs Ib -II AJCC).

## Results

The prevalence of distress, anxious and depressive symptoms was detected in 8-44% of the sample. These range is formed by: distress (44,1%), anxious symptoms (25%) and depression symptoms (8,8%) measured both with HADS, while we used the Montgomery-Asberg Depression Rating Scale to evaluated depression. Globally, high values at the SF-36 test were demonstrated, reporting a good quality of life in lower stages AJCC melanoma patients.

The Brief Cope measured styles of coping that is a psychological construct that express the way to face problems and stressful events in our life. Denial coping style resulted significantly correlated with distress, anxious and depressive symptoms and worse quality of life. On the other hand, a bad quality of life seems to influence negatively the psychological status of patients. Of them, who used active coping was more able to face the pathology and reported better psychological arrangement in their life.

Comparing the two groups (0-Ia vs Ib-II AJCC) about levels of distress, anxious and depressive symptom, coping styles and quality of life we found no statistically significant differences regarding psychological distress, measured with the Distress Thermometer and there were no statistically significant differences for anxious or depressive symptoms, measured with HADS. Quality of life, measured with SF-36, revealed no significant differences between the two groups. There were no statistically significant differences between the two groups with regard to coping styles in the majority of subscales of the Brief Cope questionnaire.

## Conclusions

This work shows the presence of psychological distress also in first stages melanoma patients population, suggesting the importance of psychological screening also in low risk melanoma patients. Moreover the findings corroborates that subjective factors, as the way of facing the pathology, are more associated with the adjustment of melanoma survivors than objective medical ones

